# How often do we need offset stems for revision total knee arthroplasty? About a consecutive series of 789 knees

**DOI:** 10.1051/sicotj/2023012

**Published:** 2023-05-29

**Authors:** Angelo V. Vasiliadis, Timothy Lording, Robin Canetti, Elvire Servien, Sébastien Lustig, Cécile Batailler

**Affiliations:** 1 Orthopaedics Surgery and Sports Medicine Department, FIFA Medical Center of Excellence, Croix-Rousse Hospital, Lyon University Hospital 69004 Lyon France; 2 Orthopedic Department, General Hospital of Thessaloniki “Papageorgiou” 56403 Thessaloniki Greece; 3 Melbourne Orthopaedic Group 33 The Avenue Windsor VIC 3181 Australia; 4 LIBM – EA 7424, Interuniversity Laboratory of Biology of Mobility, Claude Bernard Lyon 1 University 69100 Lyon France; 5 University Lyon, Claude Bernard Lyon 1 University, IFSTTAR, LBMC UMR_T9406 69622 Lyon France

**Keywords:** Total knee arthroplasty, Revision, Straight stem, Offset stem, Metaphyseal fixation

## Abstract

*Introduction*: This study aimed to determine the incidence of offset stem usage in revision total knee arthroplasty (rTKA), and to assess the necessity for their use with the femoral and tibial components. *Methods*: This retrospective radiological study included 862 patients who underwent rTKA between 2010 and 2022. Patients were divided into a non-stem group (group NS), offset stem group (group OS), and straight stem group (group SS). Two senior orthopedic surgeons evaluated all the post-operative radiographs of the group OS to assess the necessity of offset use. *Results*: In total, 789 patients met all eligibility inclusion criteria and were reviewed (305 males (38.7%)) with a mean age of 72.7 ± 10.2 years old [39; 96]. Eighty-eight (11.1%) patients had undergone rTKA with offset stems (34 tibia, 31 femur, 24 both) and 609 (70.2%) with straight stems. The tibial and femoral stems were diaphyseal of over 75 mm in 83 revisions (94.3%) for group OS and 444 revisions (72.9%) for group SS (*p* < 0.001). Offset in the tibial component was located medially in 50% of rTKA, while the offset in the femoral component was placed anteriorly in 47.3% of the rTKA. Assessment by the two independent senior surgeons found stems were only necessary in 3.4% of cases. Offset stems were only required for the tibial implant. *Discussion*: Offset stems were used in 11.1% of revision total knee replacements, however, they were deemed necessary in 3.4% and for the tibial component only.

## Introduction

While the goals of revision total knee arthroplasty (rTKA) are the same as primary total knee arthroplasty (TKA), it remains a challenging and more complex procedure due to the potential of bone defects and soft tissue insufficiency [1, 2], which often requires increased fixation and constraint [3, 4]. Possible options include cement filling, metal augments, structural bone grafts, metaphyseal sleeves/cones, and intramedullary stems, with or without offsets [3, 5]. Stems are required in most revision TKA to improve mechanical stability by resistance to shear stress and decreased micromotion [1]. However, they also have disadvantages, such as stress shielding, loosening, and stem tip pain, which may be associated with stem length and positioning [1, 6].

A valuable option to avoid the possible negative consequences of straight stems is using stems with an offset. Offset stems improve the ability to achieve maximal anatomical coverage without overhang or cortical impingement. In addition, offset stems can assist implant alignment on the metaphysis in the coronal and sagittal plane and balance the flexion and extension gaps by effectively moving the implants [1, 3, 7]. However, the current trend is towards decreased stem length and improved metaphyseal (zone 2 [8]) fixation with a cone or sleeve. This option could avoid the need for an offset, which requires additional tools and remains a potential weak point in the implant.

The primary objective of this study was to provide an estimate of the incidence of the use of offset stems in rTKA performed in a high-volume arthroplasty referral institution. The secondary objective was to retrospectively assess the necessity for an offset stem in the same series.

## Materials and methods

### Patients

**Figure 1:**
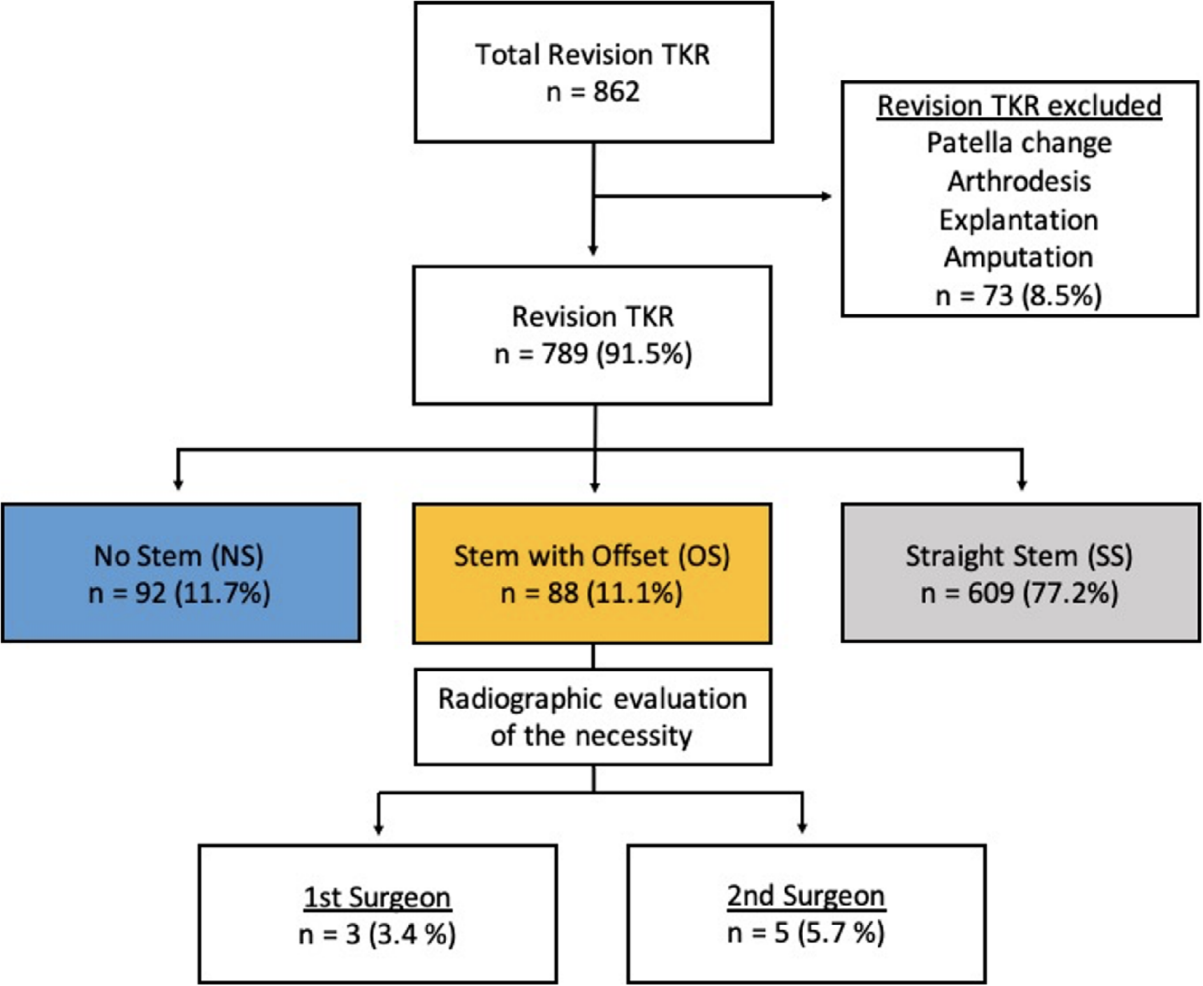
Study patient enrolment flowchart.

This was a retrospective radiological study of prospectively collected data in a high-volume arthroplasty referral institution. Inclusion criteria were rTKA using tricompartimentale prosthesis, regardless of the etiology and constraint required at revision. Three groups were identified based on the use and type of stem. In group NS (no stem), no stem was used for either the femoral or tibial implants. In group OS (offset stem), a stem with offset was used for at least one component. In group SS (straight stem), a straight stem was used for at least one component. Between January 2010 and May 2022, 862 patients underwent rTKA. Of these patients, 789 patients (305 males and 484 females), with a mean age of 72.7 ± 10.2 years old [39; 96] matched the eligibility criteria and were included in the analysis. Overall, the most common cause of revision TKR was infection (31.8%), followed by aseptic loosening (28.6%) and malposition of the implants (13.2%) (Table 1 and Figure 1).

**Table 1:** Patients’ characteristics and perioperative data of the study population.

	Group NS	Group OS	Group SS	Total	*p*
	No stem	Offset stem	Straight stem	(*n* = 789)	
	(*n* = 92)	(*n* = 88)	(*n* = 609)		
Gender, *n* (%)					0.375
Male	36 (39.1)	28 (31.8)	241 (39.6)	305 (38.7)	
Female	56 (60.9)	60 (68.2)	368 (60.4)	484 (61.3)	
Age, years, mean (±SD)	74.3 (11.4)	68.7 (7.9)	73.1 (10.6)	72.7 (10.2)	<0.001#,*
Etiology of revision, *n* (%)					0.052
Periprosthetic fracture	0 (0)	0 (0)	19 (3.1)	19 (2.4)	
Infection	31 (33.7)	20 (22.7)	200 (32.8)	251 (31.8)	
Aseptic loosening	29 (31.6)	32 (36.4)	165 (27.1)	226 (28.6)	
Malposition	9 (9.8)	11 (12.5)	84 (13.8)	104 (13.2)	
Laxity	11 (12)	6 (6.8)	53 (8.7)	70 (8.9)	
Stiffness	4 (4.3)	6 (6.8)	51 (8.4)	61 (7.7)	
Pain	4 (4.3)	7 (8)	21 (3.5)	32 (4.1)	
PE wear	0 (0)	0 (0)	3 (0.5)	3 (0.4)	
Patella issues	0 (0)	0 (0)	2 (0.3)	2 (0.3)	
Allergy	4 (4.3)	6 (6.8)	11 (1.8)	21 (2.7)	
Constraint, *n* (%)					<0.001
No constraint	92 (100)	8 (9.1)	162 (26.6)	262 (33.2)	
CCK	0 (0)	79 (89.8)	174 (28.6)	253 (32.1)	
Hinged	0 (0)	1 (1.1)	273 (44.8)	274 (34.7)	
TT osteotomy, *n* (%)	0 (0)	21 (23.9)	148 (24.3)	169 (21.4)	<0.001#,^
Tibial stem, *n* (%)	0 (0)	88 (100)	609 (100)	697 (88.3)	<0.001#,^
Femoral stem, *n* (%)	0 (0)	87 (98.9)	497 (81.6)	584 (74)	<0.001#,^
Length of stem					<0.001
No stem	92 (100)	0 (0)	0 (0)	92 (11.7)	
Metaphyseal, 30–75 mm	0 (0)	5 (5.7)	165 (27.1)	170 (21.5)	
Diaphyseal, > 75 mm	0 (0)	83 (94.3)	444 (72.9)	527 (66.8)	

Abbreviations: CCK, constrained condylar knee; TT, tibial tuberosity.

# A significant correlation between group NS versus group OS.

* A significant correlation between group OS versus group SS.

^ A significant correlation between group NS versus group SS.

### Data assessment

All surgeries were performed by four senior orthopedic surgeons specializing in adult reconstructive knee surgery. To determine the appropriate component size and positioning, all patients underwent pre-operative radiographic evaluation with templating. Patient information was abstracted from a prospective registry and is summarized in Table 1. Tibial tubercle osteotomy was performed for difficult exposure, component extraction, or patellar maltracking. A tibial offset was used when a straight stem did not allow optimal tibial coverage with a stem longer than the previously used stem. A femoral offset was used when a straight stem did not restore the posterior or anterior offset or led to a medial or lateral overhang. Anteroposterior and lateral radiographs were performed post-operatively in all the patients.

**Figure 2:**
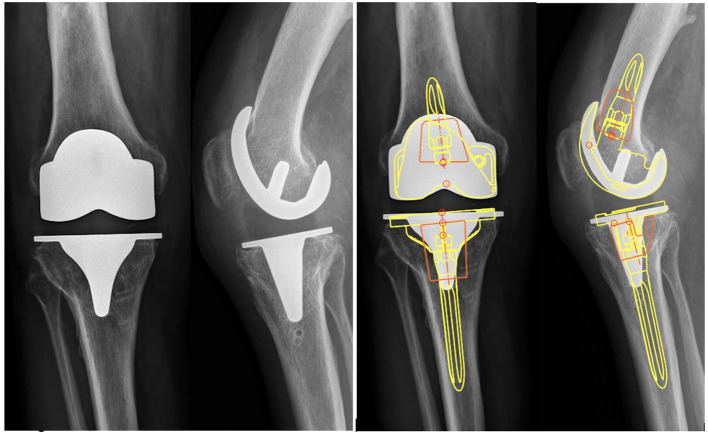
Assessment of the necessity of offset stems on the preoperative radiographs with the Traumacad^®^ software (Yellow: implants and stem; Orange: metaphyseal cones).

Two orthopedic surgeons independently analyzed the post-operative radiographs for the necessity of offset stems. Using Traumacad^®^ software (Traumacad^®^, Petach-Tikva, Israel) femoral and tibial components were templated for size and positioning, and stems were templated for length, diameter, and need for offset. An offset stem was deemed necessary retrospectively when it was not possible to use a straight stem while respecting the following criteria: (1) adequate coverage of the epiphysis (the largest possible implant without overhang); (2) the shortest stem possible (75 or 100 mm for standard cases, or a stem with sufficient length to bridge a weak zone); (3) an absence of malalignment due to the stem positioning (Figure 2). In case of discrepancy between the surgeons, a third experienced surgeon assessed the case and took the final decision.

### Statistical analysis

Collected data were analyzed with SPSS (Version 24.0). Continues variables were expressed as mean ± sd [range]; categorical variables as percentages. The Kolmogorov–Smirnov test was utilized for normality analysis. Mann–Whitney *U*-test was utilized for the comparison of the continuous variables in the independent samples, for non-normal distribution, in a population divided into two categories. The Kruskal–Wallis test was utilized for the comparison of continuous variables in the independent samples, for non-normal distribution, in a population divided into more than 2 categories. Pearson-*χ*^2^ (cross-tabulation) was utilized for the comparison of categorical variables. The level of significance was set at *p* < 0.05.

## Results

**Figure 3:**
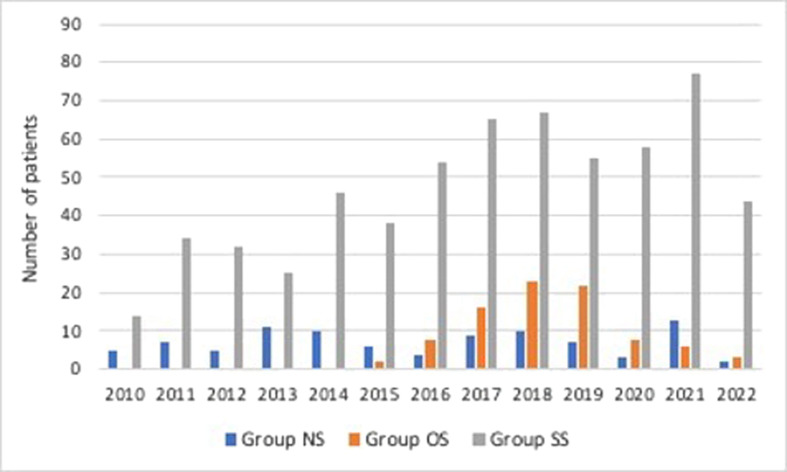
Distribution of the number of patients operated per year.

The incidence rate for offset stem use was 11.1% (*n* = 88). The distribution of offset stem over time is displayed in Figure 3. Regarding the constraint choice for the revision prosthesis, a higher constraint prosthesis design was reported in the group OS and group SS compared with group NS (*p* < 0.001). The distribution pattern for constrained knees was varus-valgus constrained (VVC) TKA in 89.8% (*n* = 79) and hinge TKA in 1.1% (*n* = 1) for group OS compared to 28.5% VVC (*n* = 174) and 44.8% hinged (*n* = 273) for group SS (*p* < 0.001) (Table 1). A tibial stem was used in all rTKA in group OS and SS, whereas femoral stems were used in 98.9% of rTKA in group OS and 81.6% of rTKA in group SS. The tibial and femoral stems were metaphyseal (length 30–75 mm) for 5.7% (*n* = 5) and 27.1% (*n* = 165) for group OS and group SS, respectively, while stems were diaphyseal (length > 75 mm) in 94.3% (*n* = 83) for group OS and 72.9% (*n* = 444) for group SS (Table 1).

**Figure 4:**
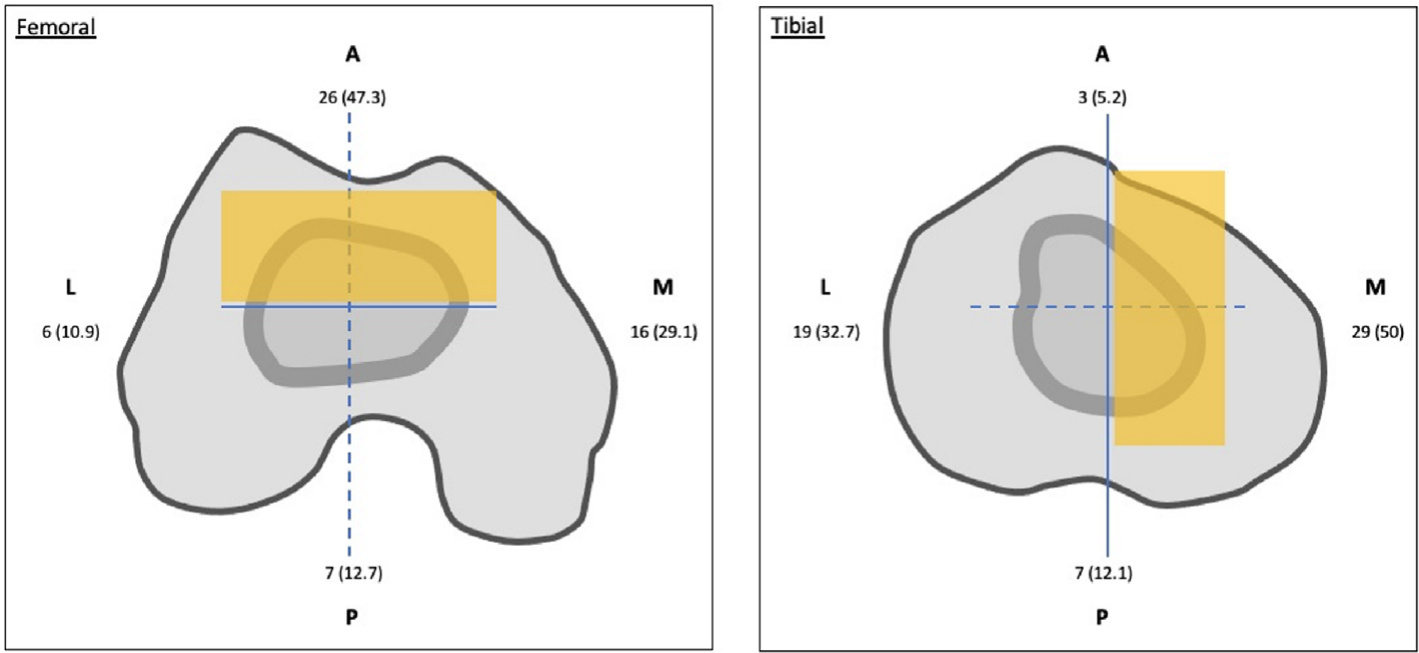
The percentage predominance of stems with offset according to the direction in femoral and tibial medullary canal. Anterior and medial position, which indicated with orange, was the main location of most stems with offset for femoral and tibial medullary canal, respectively.

In group OS, femoral stems with offset were chosen in 31 (35.2%) out of 88 cases and tibial stems with offset were used in 34 (38.6%) out of 88 cases, while both combinations were chosen in 24 (27.3%). Most of the offset for the femoral stem was placed in an anterior position (*n* = 26, 47.3%), while most of the offset for the tibial stem was placed in a medial position (*n* = 29, 50%) (Figure 4).

**Figure 5:**
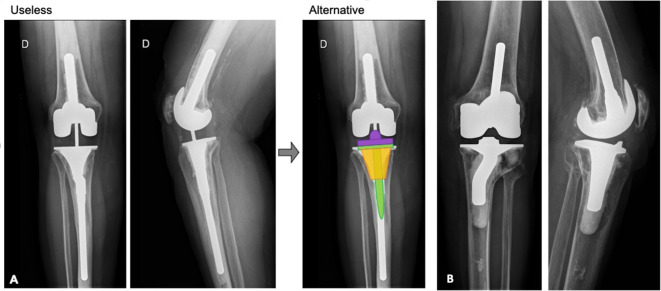
Post-operative radiographic evaluation demonstrating a not-required (A) and a required (B) stem with offset. A possible alternative was illustrated. A cone (orange) and a shorter straight stem (green) can be used to increase the contact area and compensate for metaphyseal bone loss.

Initial assessment of the radiographs by two independent senior surgeons found that only 3.4% (*n* = 3) and 5.7% (*n* = 5) of offset stems were necessary (Figure 5). After the assessment of the third surgeon, only 3.4% (*n* = 3) of offset stems were necessary. Offset stems were only required for the tibial implant when the tibial plateau was translated laterally compared to the diaphysis, secondary to tibial malunion or previous high tibial osteotomy. A femoral offset was never necessary for this series. A shorter stem or improved femoral component positioning could avoid an offset in every case.

## Discussion

The main finding of this study was that the stems with offset were used in revision TKA in 11.1% of procedures over 12 years but were only deemed necessary in 3.4% after a new assessment by experienced surgeons on postoperative radiographs.

During rTKA, using more constrained implants, such as VVC and hinged prostheses, require femoral and tibial stems [3, 9]. Stems have a load-sharing capability, increase initial femoral and tibial component stability, and aid the remaining damaged or absent metaphyseal bone to deal with excessive stress [10]. Its main role is to engage healthy bone and bypass any metaphyseal or/and diaphyseal defects [1, 10–12]. Previously, stems were frequently long to improve implant fixation. With the long stems, using an offset enhances the ability to achieve maximal anatomical coverage without overhang or cortical impingement. Previous studies have reported advantages of the use of stem with offset in rTKA (Table 2) [3, 13–16]. This surgical strategy of a long stem with offset was favored previously in the department and explains the rate of 11% of offset stems. As described in the literature (Table 2), we used offset stem to improve the bone coverage with long stems or when there was a malunion with a diaphysis not aligned with the metaphysis.

**Table 2:** Published outcomes following revision TKA with the use of offset stems on the femoral and tibial sides.

Author	Years	rTKA	Mean age	Etiology	Straight/offset stems	Follow-up	Outcomes/Conclusion
Present study	2010–2022	789	72.7 years	Infection (31.8%)	609/88	59.3 months	Offset stems are occasionally (11%) in rTKA and probably overestimate regarding post-operative radiographic evaluation
				Aseptic loosening (28.6%)			
				Malposition (13.2%)			
Rosso et al. [13]	2008–2016	53	71.5 years	Aseptic loosening (41.5%)	37/16	56.6 months	A stepwise approach may achieve good clinical and radiological outcomes
				Infection (30.2%)			
				Instability (9.4%)			
Crawford et al. [14]	2005–2013	278	67 years	Aseptic loosening (47%)	241/37 (tibial)	6 years	Modular revision systems improve clinical outcomes and provide good survivorship
				Instability (13.2%)			
				Polyethylene wear (22%)			
Brilhault and Ries [3]	1998–2005	126	57.9 years	n/a	91/35 (femoral)	4.5 years	Offset femoral stem increases the resulting PCO and improves alignment
Sah et al. [15]	1998–2003	88	68.8 years	Aseptic loosening (52%)	62/26 (femoral)	65 months	Hybrid stem fixation contributes to a durable fixation and reduces stem tip pain
				Infection (17%)	24/64 (tibial)		
				Instability (9%)			
Nakasone et al. [16]	2001–2003	52	64.7 years	Infection (31%)	0/52	n/a	Diaphyseal uncemented offset stems facilitate accurate alignment for both femoral and tibial components
				Stiffness (23%)			
				Aseptic loosening (17%)			

rTKA: revision total knee arthroplasty; PCO: posterior condylar offset; n/a: not applicable.

However, there are possible disadvantages to the use of long stems, which can be avoided with metaphyseal fixation [8]. These include stress shielding along the length of the stem, the potential of stem tip pain with uncemented stems, the risk of loosening, and periprosthetic fracture [10–12, 17]. Biomechanical studies have shown that stems of 70 mm in length carried up to 38% of the axial load, minimizing the load of the metaphysis [1, 18]. In comparison, stems of 150 mm in length produced stress shielding in the proximal part of the tibia, resulting in excessive strain at the stem tip [1]. Using computer-assisted software in a cadaveric study, Gobba et al. demonstrated that tibial stems of 200 mm in length might lead to valgus malalignment and malposition of the tibial component [19]. When the surgeon is faced with these situations, the solution can be to use a shorter, cemented tibial stem with metaphyseal augments such as cones or sleeves. These alternatives are usually indicated for reconstructing large metaphyseal rim defects with the significant cancellous bone loss [5, 20]. These methods can provide a stable construct and have shown excellent osseointegration and functional outcomes at mid-term follow-up [20, 21]. Different types of augments and stems can be used to fix rather than bypass the problem [5]. Although the combined use of augments and stems is mandatory to provide a stable fixation in significant metaphyseal bone loss, sleeves, and cones can provide good metaphyseal fixation, which may permit the use of a shorter stem, and reduce the degree of canal filling [22–24]. This evolution in surgical philosophy explains the low rate of only 3.4% where an offset was considered necessary.

In some cases, an offset stem was still indicated. For all these cases, the deformity needing an offset was on the tibial side, with a lateral displacement of the tibial plateau compared to the diaphysis. This deformity was due to malunion or high tibial osteotomy by a lateral closing wedge. In a cadaveric study, Hicks et al. tried to determine the relationship between the tibial plateau and the intramedullary canal of the tibia. They noted that the center of the intramedullary canal was relatively anterior and medial to the tibial plateau, indicating the need for pre-operative templating in revision cases using long stems [25]. In the current study, in half of the patients, the axis of the tibial shaft was located medially to the center of the tibial plateau when the tibial component was positioned, for whom a medially offset was placed. For the femoral implant, the offset aimed to displace the femoral implant posteriorly to preserve the posterior offset. Nevertheless, the femoral implant can be well positioned with a short femoral stem without offset. The optimal indications of offset stems remain tibial malunion or high tibial osteotomy by a lateral closing wedge. An indication, not reported in this study, is the needing to bypass a diaphysis fracture or bone defect with a long stem.

Our study should be interpreted in light of some limitations. Firstly, due to the study design’s descriptive nature, it cannot imply causality or provide explanations for unexpected findings. Furthermore, no clinical outcomes or survival were assessed. However, this study aimed to analyze and improve our preoperative planning for rTKA. Finally, the patient population at this academic orthopedic institution may not accurately represent the general worldwide population, and caution should be taken in extrapolating these results.

## Conclusions

Stems with offset were used in 11.1% of rTKA but were deemed necessary in only 3.4% after a retrospective radiographic assessment considering the current approach of RTKA with a zonal fixation concept. With metaphyseal fixation, the requirement for offset stems is very low, except in some post-traumatic or post-osteotomy cases on the tibial side.
